# Key Inflammatory Processes in Human NASH Are Reflected in Ldlr^−/−^.Leiden Mice: A Translational Gene Profiling Study

**DOI:** 10.3389/fphys.2018.00132

**Published:** 2018-02-23

**Authors:** Martine C. Morrison, Robert Kleemann, Arianne van Koppen, Roeland Hanemaaijer, Lars Verschuren

**Affiliations:** ^1^Department of Metabolic Health Research, The Netherlands Organization for Applied Scientific Research (TNO), Leiden, Netherlands; ^2^Department of Vascular Surgery, Leiden University Medical Center, Leiden, Netherlands; ^3^Department of Microbiology and Systems Biology, The Netherlands Organization for Applied Scientific Research (TNO), Leiden, Netherlands

**Keywords:** liver, NASH, inflammation, molecular, gene expression, translational, mouse, human

## Abstract

**Introduction:** It is generally accepted that metabolic inflammation in the liver is an important driver of disease progression in NASH and associated matrix remodeling/fibrosis. However, the exact molecular inflammatory mechanisms are poorly defined in human studies. Investigation of key pathogenic mechanisms requires the use of pre-clinical models, for instance for time-resolved studies. Such models must reflect molecular disease processes of importance in patients. Herein we characterized inflammation in NASH patients on the molecular level by transcriptomics and investigated whether key human disease pathways can be recapitulated experimentally in Ldlr^−/−^.Leiden mice, an established pre-clinical model of NASH.

**Methods:** Human molecular inflammatory processes were defined using a publicly available NASH gene expression profiling dataset (GSE48452) allowing the comparison of biopsy-confirmed NASH patients with normal controls. Gene profiling data from high-fat diet (HFD)-fed Ldlr^−/−^.Leiden mice (GSE109345) were used for assessment of the translational value of these mice.

**Results:** In human NASH livers, we observed regulation of 65 canonical pathways of which the majority was involved in inflammation (32%), lipid metabolism (16%), and extracellular matrix/remodeling (12%). A similar distribution of pathways across these categories, inflammation (36%), lipid metabolism (24%) and extracellular matrix/remodeling (8%) was observed in HFD-fed Ldlr^−/−^.Leiden mice. Detailed evaluation of these pathways revealed that a substantial proportion (11 out of 13) of human NASH inflammatory pathways was recapitulated in Ldlr^−/−^.Leiden mice. Furthermore, the activation state of identified master regulators of inflammation (i.e., specific transcription factors, cytokines, and growth factors) in human NASH was largely reflected in Ldlr^−/−^.Leiden mice, further substantiating its translational value.

**Conclusion:** Human NASH is characterized by upregulation of specific inflammatory processes (e.g., “Fcγ Receptor-mediated Phagocytosis in Macrophages and Monocytes,” “PI3K signaling in B Lymphocytes”) and master regulators (e.g., TNF, CSF2, TGFB1). The majority of these processes and regulators are modulated in the same direction in Ldlr^−/−^.Leiden mice fed HFD with a human-like macronutrient composition, thus demonstrating that specific experimental conditions recapitulate human disease on the molecular level of disease pathways and upstream/master regulators.

## Introduction

Non-alcoholic fatty liver disease (NAFLD) is one of the most important causes of chronic liver disease worldwide (Wong et al., 2015; Younossi et al., 2017). The prevalence of NAFLD is rising in close association with the increasing prevalence of obesity, insulin resistance, and dyslipidemia, all of which are risk factors for NAFLD (Siddiqui et al., 2015; Chang et al., 2016; Dongiovanni et al., 2016; Katsiki et al., 2016). NALFD encompasses a spectrum of liver disease: ranging from the relatively benign hepatic steatosis, which is characterized by the accumulation of lipids in the liver, to non-alcoholic steatohepatitis (NASH), the progressive form of NAFLD.

NASH is characterized by the presence of hepatocellular damage and inflammation (Rinella, 2015), which in concert can drive the development of liver fibrosis (Fujii and Kawada, 2012), the strongest predictor of NAFLD-related mortality (Angulo et al., 2015; Ekstedt et al., 2015). The hepatic inflammatory response in NASH is poorly characterized on the molecular level and thought to originate from a combination of various chronic pro-inflammatory triggers (Tilg and Moschen, 2010). In addition to direct lipotoxicity resulting from the build-up of pro-inflammatory lipid species in the liver (such as free fatty acids, diglycerides, ceramides, and free cholesterol) (Alkhouri et al., 2009), the inability of hepatocytes to cope with an increased metabolic load is thought to lead to ER stress, metabolic dysfunction and production of reactive oxygen species, which in turn can contribute to exacerbation of the hepatic inflammatory response (Takaki et al., 2014). On top of that, it is thought that extrahepatic pro-inflammatory signals from the adipose tissue and the gut can drive hepatic inflammation in NASH (Tilg and Moschen, 2010; Takaki et al., 2014).

The exact molecular events in liver that contribute to this chronic inflammatory condition are not well-characterized, and much remains unknown about the nature of these molecular inflammatory processes in NASH. While a number of studies has explored genome-wide hepatic gene expression in NASH patients (Younossi et al., 2005; Zhang et al., 2012; Moylan et al., 2014; Arendt et al., 2015; Teufel et al., 2016) none of these has focused on unraveling the inflammatory response. Since studies based on human liver biopsy material generally do not allow in-depth mechanistic studies or time-resolved investigation of disease progression, pre-clinical disease models are key to the development of a deeper mechanistical understanding of pathogenesis and are required for testing of new therapeutic interventions (Hebbard and George, 2011). To study mechanisms of disease development in pre-clinical models, it is critical that the model employed is reflective of human disease processes.

A wide variety of animal models for NASH is available, each with their own specific advantages and disadvantages (Ibrahim et al., 2016; Jacobs et al., 2016). The translational value of these experimental models is often judged on basis of histopathological features but not on the molecular pathophysiological level, i.e., whether they recapitulate the disease pathways that are evoked in NASH patients. Hence, the translational value of most models is currently under debate, the more so because many models are not sufficiently validated. A recent study compared different animal models with the full spectrum of NAFLD patients using gene profiling and concluded that none of the investigated animal models mimics the complete spectrum of molecular processes involved in humans (Teufel et al., 2016). However, it has been shown that diet-inducible models showed at least some similarities in the development of disease. Therefore this study and other reports (Hebbard and George, 2011; Mulder et al., 2016) suggest that diet-induced models may best reflect sub-processes of human disease phenotypes and underlying pathogenesis.

In the current study we characterized specifically the sub-process of inflammation in human NASH and evaluated the translational value of the high-fat-diet (HFD)-fed Ldlr^−/−^.Leiden mouse with regards to the hepatic inflammatory response in NASH. This NASH model was not included in the comparison of hepatic gene expression in murine and human NASH described above (Teufel et al., 2016). Ldlr^−/−^.Leiden mice display phenotypical and histopathological characteristics of NASH patients when fed a human like-HFD without requiring amino acid and choline deficiency or supraphysiologic levels of cholesterol in the diet (Liang et al., 2014; Morrison et al., 2016; van Koppen et al., 2018). More specifically, they develop NASH in the context of an obese phenotype with hypercholesterolemia, hypertriglyceridemia, and hyperinsulinemia as observed in many patients (Anderson and Borlak, 2008; Loomba and Sanyal, 2013). Histopathologically, these mice show presence of micro- and macrovesicular steatosis in the liver, hepatocellular hypertrophy with hepatocellular disintegration and sporadic ballooning, lobular inflammation (mixed-cell inflammatory infiltrates) and marked hepatic fibrosis that progresses with prolonged HFD treatment. In a recent comparison of NASH-induced regulation of hepatic gene expression (using an unbiased approach), HFD-fed Ldlr^−/−^.Leiden mice were found to recapitulate many of the gene expression changes observed in human NASH (van Koppen et al., 2018). The current study focuses specifically on the inflammatory component of NASH, first defining the main molecular inflammatory processes present in human NASH using available published information and subsequently exploring the representation of these processes in HFD-fed Ldlr^−/−^.Leiden mice.

## Materials and methods

### Human hepatic gene expression dataset

For the investigation of molecular inflammatory processes in human NASH, samples were selected from a published dataset accessible at the NCBI Gene Expression Omnibus (GEO) database, accession GSE48452. This dataset originates from a study on DNA methylation patterns in NASH patients (Ahrens et al., 2013) that included morbidly obese patients with biopsy-confirmed NAFLD pre- and post-bariatric surgery, as well as healthy controls. In this study, gene expression levels were measured using Affymetrix Human Gene 1.1 ST array (Affymetrix Inc., Santa Clara, California, USA). For the purpose of the present study, we selected the pre-bariatric surgery samples from NASH patients (*n* = 17) and compared them with the healthy controls (*n* = 12) (see sample list in Supplemental Table 1). The probe-level, background-subtracted, expression values were used as input for lumi package (Du et al., 2008) of the R/Bioconductor (http://www.bioconductor.org; http://www.r-project.org) to perform quality control and quantile normalization. Differentially expressed probes were identified using the limma package of R/Bioconductor (Wettenhall and Smyth, 2004), calculated values of *P* < 0.01 were used as threshold for significance.

### Murine hepatic gene expression dataset

Murine gene expression data was obtained from a previously published study (van Koppen et al., 2018) and the dataset is accessible at the NCBI GEO database via accession number GSE109345. Detailed methods of tissue collection and processing as well as next generation sequencing (NGS) and statistical analysis are described in van Koppen et al. (2018). Briefly, *n* = 15 Ldlr^−/−^.Leiden mice (94% C57BL/6J background, 6% 129S1/SvImJ background) were fed an energy-dense high-fat diet (HFD; 45 kcal% fat from lard, 17 kcal% sucrose) for 24 weeks to induce NASH (steatosis, inflammation, early fibrosis) in the context of obesity, hyperlipidemia and hyperinsulinemia. Hepatic gene expression dataset of HFD-treated mice was generated and data were expressed relative to age-matched chow-fed controls (*n* = 15).

### Data- and statistical analysis

Differentially expressed probes (from human dataset) and counts (from mouse dataset) confined with the statistical cut-off were used as input for pathway analysis through Ingenuity Pathway Analysis (IPA) suite (www.ingenuity.com, accessed 2017) as described previously (Morrison et al., 2015; Mulder et al., 2016; van Koppen et al., 2018). An upstream regulator analysis, which is part of IPA, predicts the activation state of a protein, enzyme or transcription factor based on the expression pattern of the genes downstream of this factor. The z-score indicates the predicted activation state of a transcription factor or key regulator: *z* ≤ −2 indicates relevant inhibition (shown in green), *z* ≥ 2 indicates relevant activation (shown in red). Pathway-based overlap analysis was performed by using Venny 2.1 (Oliveros, 2007) and heatmaps were generated using a web-based tool (Babicki et al., 2016). The genes that belong to significantly regulated inflammatory canonical pathways were visualized using Neo4J (Neo4j, Inc, San Mateo, CA, USA) a graph database with query-based calculations.

## Results

### Key inflammatory processes and regulators in NASH liver biopsies

To define the major inflammatory processes that are modulated during NASH development, gene expression profiles in liver biopsies from NASH patients were compared to those of healthy control livers using a published human dataset (GSE48452). In total 12 healthy controls and 17 patients with biopsy-proven NASH were analyzed. At a statistical cut-off of *P* < 0.01, 972 genes were differentially expressed between the two groups, i.e., 519 genes were upregulated and 453 genes were downregulated in NASH livers relative to healthy controls. All these NASH-associated genes were used as input for pathway analysis, which integrates the expression of a multitude of genes in pre-defined biological and disease-associated pathways thus allowing summation of multiple (small) gene expression changes to provide information on the effect on entire pathways rather than single genes.

**Figure 1:**
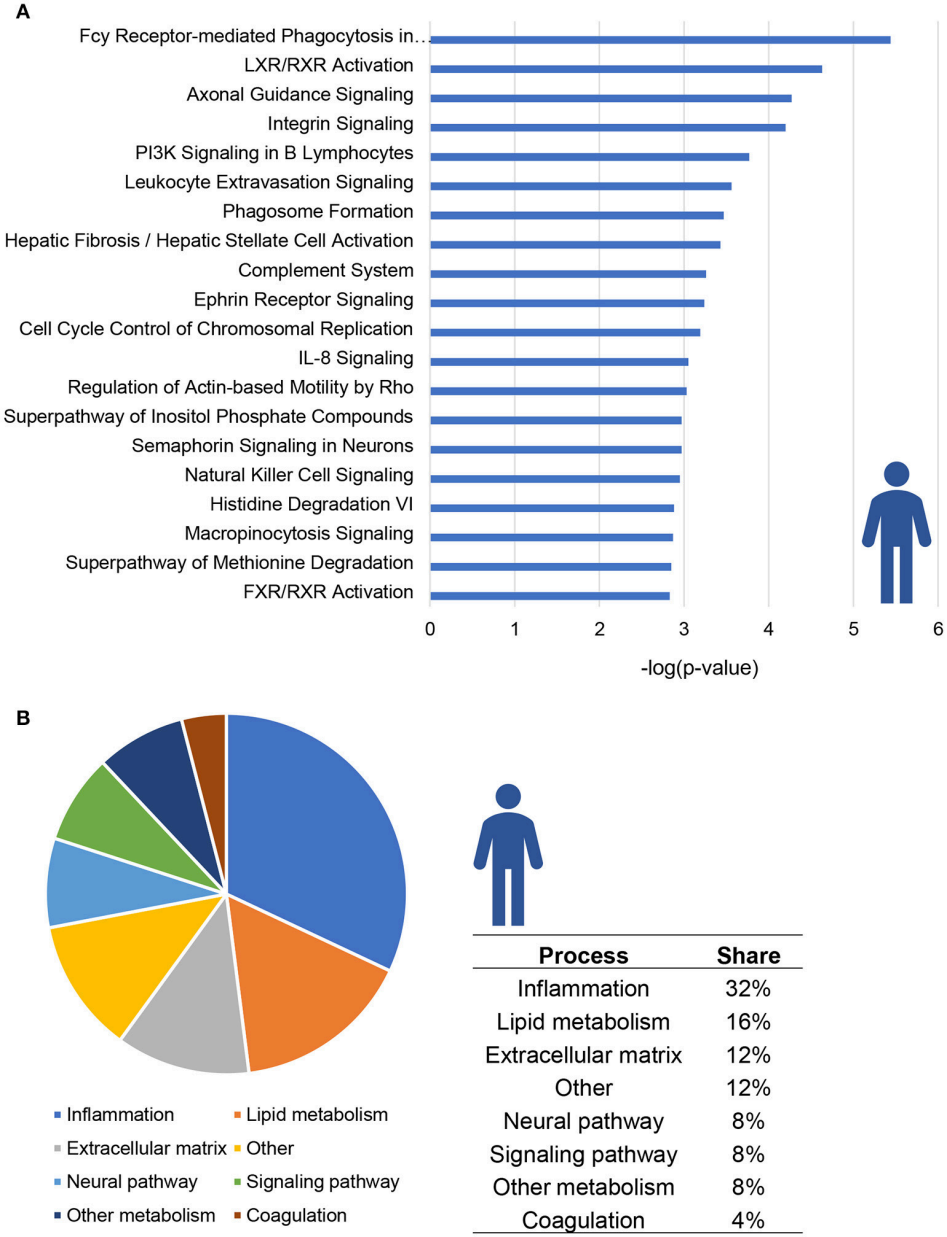
Enrichment analysis of molecular pathways in NASH patients. **(A)** Visualization of top 20 enriched canonical pathways in human NASH patients as compared to normal controls. Values are expressed as –log(*p*-value). **(B)** Circle chart which classifies canonical pathways into more general biological processes and illustrates the proportion of pathways in each of these categories.

The top 20 most significantly enriched pathways are visualized in Figure 1A; this top 20 includes canonical pathways involved in various (patho)physiological processes such as lipid metabolism, inflammation and hepatic fibrosis. When taking all significantly enriched canonical pathways [−log(*P*-value) > 2] into account, the majority of pathways appear to be related to inflammation (32%) while other pathways are related to lipid metabolism (16%) or extracellular matrix remodeling (12%; Figure 1B).

**Figure 2:**
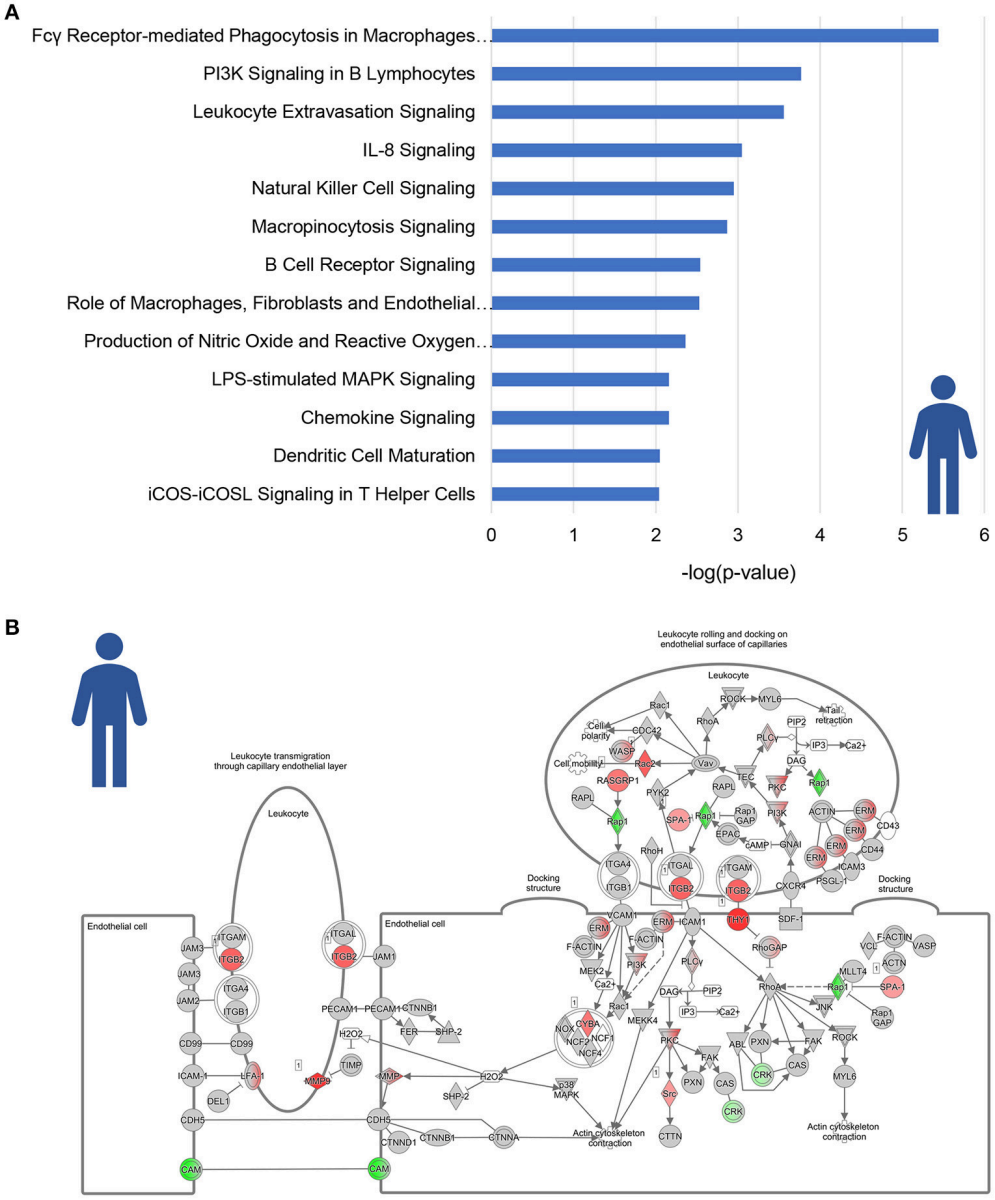
Enrichment analysis of inflammatory pathways in NASH patients. **(A)** Visualization of all inflammation-related canonical pathways in human NASH patients as compared to normal controls. Values are expressed as –log(*p*-value). **(B)** Visualization of the expression change in NASH patients as compared to normal controls for the canonical pathway “Leukocyte Extravasation Signaling.” Red color indicates significant upregulated genes and green color indicates significant downregulated genes.

A total of 13 pathways was related to inflammation, among which “Fcγ Receptor-mediated Phagocytosis in Macrophages and Monocytes,” “PI3K signaling in B Lymphocytes” and “Leukocyte Extravasation Signaling” (Figure 2A). Subsequent refined analysis of the genes that are part of these pathways showed that the expression of the majority of the individual genes is significantly upregulated in NASH patients relative to controls. The consistency of this upregulation is exemplified for the pathway “Leukocyte Extravasation Signaling” (Figure 2B).

We subsequently analyzed inflammatory processes in NASH in more detail by performing an upstream regulator analysis. This analysis predicts the activation state (indicated by a positive/negative z-score for activation/inhibition) of upstream regulators (e.g., transcription factors and cytokines) in NASH livers relative to healthy controls. Table 1 shows the inflammation-related upstream regulators that were significantly activated (in red) or inhibited (in green) in NASH patients. We observed an activation of classical regulators of inflammation such as the cytokines TNF (Tumor necrosis factor alpha), CSF2 (Granulocyte-macrophage colony-stimulating factor) and TGFB1 (Transforming growth factor beta 1) as well as factors that can indirectly affect inflammatory processes, such as ESR2 (Estrogen receptor 2), and PLG (Plasminogen). In addition, a known inhibitor of inflammation AHR (Aryl hydrocarbon receptor) (Li et al., 2011) was predicted to be inactive in NASH relative to healthy controls.

**Table 1:** Significantly modulated inflammation-related upstream regulators in human NASH vs. control.

	**NASH vs. CTRL**	
**Upstream regulator**	**Activation z-score**	***p*****-value**
TNF	2.426	0.00000011
CEBPA	2.149	0.0000251
CSF2	3.144	0.0000448
IGF1	3.918	0.000118
SP1	3.14	0.000154
IL5	1.86	0.000213
DSP	−2.219	0.000327
TGM2	2.18	0.000372
ERG	3.035	0.000386
TGFB1	2.64	0.00051
ERBB2	3.393	0.00064
SREBF2	3.115	0.000703
PARP2	1.987	0.000706
ATP7B	2.646	0.000872
MLXIPL	2.198	0.00113
RUNX3	−1.982	0.00125
SNAI1	2.219	0.00183
ESR2	2.316	0.00254
EGR1	2.381	0.00264
CREBBP	2.266	0.00265
PLG	2.121	0.00298
FGF2	2.203	0.00317
ETS2	2.158	0.0051
AHR	−3.035	0.00528
RHOA	2.63	0.00694
TET2	2.111	0.00708
GLI1	2.072	0.00841

*The z-score indicates the predicted activation state of a transcription factor or key regulator: z ≤ −2 indicates relevant inhibition (shown in green), z ≥ 2 indicates relevant activation (shown in red). The p-value indicates significant enrichment of the genes downstream of a regulator (p < 0.01 was considered statistically significant)*.

### Key inflammatory processes and regulators in murine NASH development

**Figure 3:**
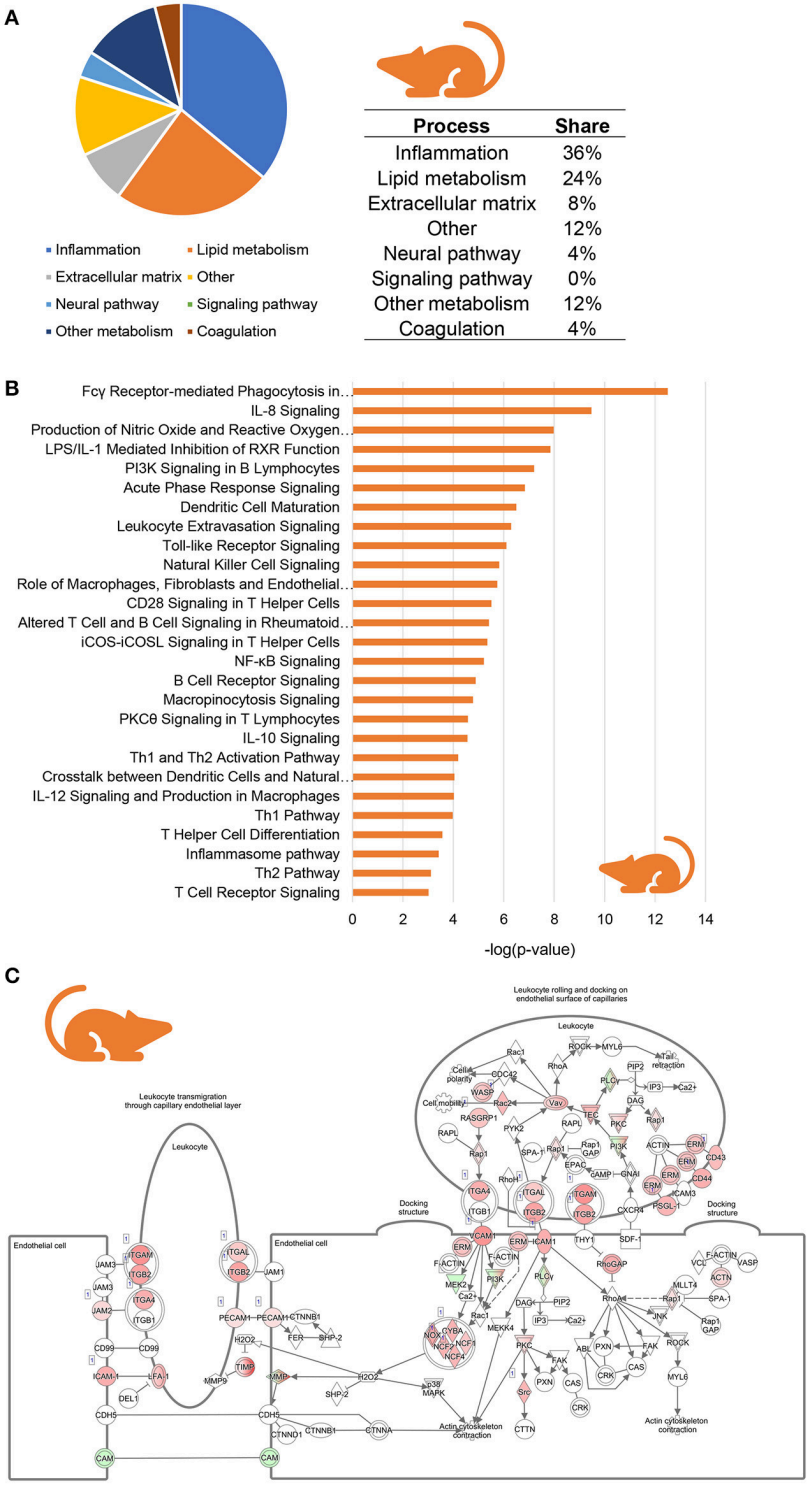
Enrichment analysis of molecular pathways in HFD-fed Ldlr^−/−^.Leiden mice. **(A)** Circle chart which classifies canonical pathways into more general biological processes and illustrates the proportion of pathways in each of these categories. **(B)** Visualization of all inflammation-related canonical pathways in HFD-fed Ldlr^−/−^.Leiden mice. **(C)** Visualization of the expression change in HFD-fed Ldlr^−/−^.Leiden mice as compared to chow controls for the canonical pathway “Leukocyte Extravasation Signaling.” Red color indicates significant upregulated genes and green color indicates significant downregulated genes.

To investigate whether similar inflammatory processes were evoked in murine NASH, we studied hepatic gene expression profiles of HFD-fed Ldlr^−/−^.Leiden mice, which display phenotypical and histopathological characteristics of NASH patients (Liang et al., 2014; Morrison et al., 2016). We compared hepatic gene expression profiles of 24-week HFD-fed mice (at the stage of hepatic steatosis, inflammation and early fibrosis) to those of age-matched healthy controls (chow-fed Ldlr^−/−^.Leiden mice) using data from a recently published study (van Koppen et al., 2018, GSE 109345). In total, 2,680 genes were differentially expressed between the two groups (1,639 upregulated, 1,046 downregulated; *P* < 0.001). Subsequent pathway analysis revealed that, as seen in human NASH, the majority of regulated pathways is related to inflammation (36%) while many of the other most significantly regulated pathways are involved in lipid metabolism (24%) and extracellular matrix remodeling (8%) (Figure 3A). A total of 27 pathways were related to inflammation, for instance “Fcγ Receptor-mediated Phagocytosis in Macrophages and Monocytes,” “IL-8 signaling,” “Production of Nitric Oxide and Reactive Oxygen Species in Macrophages” and “Leukocyte Extravasation Signaling” (Figure 3B). As observed in human NASH, the expression of most of the individual genes that make up these inflammatory pathways were upregulated in HFD-fed mice relative to healthy controls, as is illustrated for the “Leukocyte Extravasation Signaling” pathway (Figure 3C). To determine which inflammation-related upstream regulators are involved in the development of murine NASH, an upstream regulator analysis was performed to identify significantly activated or inhibited regulators in HFD-fed Ldlr^−/−^.Leiden mice relative to their age-matched healthy controls (shown in Table 2). This analysis revealed significant activation of many classical chemokines (e.g., CCL2, CCL5, CXCL2, CXCL3), cytokines (e.g., TNF, TGFB1, IL1B, CSF2) and transcriptional regulators of inflammation (e.g., NF-KB, STAT4, JUN) as well as factors with indirect links to inflammation (e.g., FGF2, FOXO1, PLG). Several anti-inflammatory factors were found to be significantly inactivated in NASH livers including AHR and others (e.g., IL10RA, IL1RN) which is consistent with the observations in humans.

**Table 2:** Significantly modulated inflammation-related upstream regulators in HFD-fed Ldlr^−/−^.Leiden mice vs. chow.

	**HFD vs. Chow**	
**Upstream regulator**	**Activation z-score**	***p*****-value**
TGFB1	7.849	1.96E-57
TNF	9.244	6.44E-52
IFNG	8.757	3.3E-47
TP53	2.363	9.73E-42
IL1B	7.97	4.03E-34
ERBB2	2.374	1.38E-32
IL6	4.47	1.68E-32
IL13	3.514	3.49E-30
IL10RA	−3.494	2.83E-26
IL4	3.087	3.12E-26
AHR	−3.408	1.99E-25
NFKBIA	3.976	3.41E-22
SP1	3.337	3.99E-22
STAT3	2.572	5.1E-22
CSF2	6.882	3.02E-21
IL2	5.83	1.65E-18
JUN	3.138	3.34E-18
SREBF2	−4.082	5.14E-18
IKBKB	4.584	4.38E-17
STAT1	6.505	5.96E-17
IFNB1	3.99	6.31E-17
CSF1	4.48	1.59E-16
CSF3	2.881	9.27E-16
CD44	5.442	9.32E-16
CEBPB	2.814	5.24E-15
TGM2	5.805	1.16E-14
IL1	5.181	1.66E-14
EGF	4.346	4.27E-14
IL5	4.57	4.3E-14
MYD88	6.236	4.42E-14
TLR4	6.077	9.01E-14
IL17A	4.377	3.69E-13
IKBKG	4.505	5.84E-13
IL3	3.661	1.73E-12
IGF1	3.725	7.38E-12
IL1A	5.712	1.55E-11
CREBBP	2.868	3.2E-11
STAT4	4.14	3.74E-11
TLR3	5.923	4.1E-11
FOXO1	3.171	4.13E-11
TLR9	5.532	4.79E-11
FGF2	3.295	8.43E-11
HGF	3.624	1.64E-10
EGR1	3.58	2.35E-10
CCL5	2.583	4.74E-10
NFKB1	4.307	6.73E-10
SMAD3	4.858	2.92E-09
RELA	4.325	4.43E-09
ATP7B	−3.771	4.66E-09
CXCL12	4.638	1.09E-08
IL15	3.372	1.72E-08
JUNB	2.91	5.15E-08
SPDEF	−3.725	5.64E-08
TNFSF12	4.161	7.22E-08
FGFR2	2.368	1.05E-07
TGFA	2.134	2.15E-07
LIF	2.452	4.86E-07
IL1RN	−4.697	7.80E-07
IL18	5.15	8.27E-07
IL21	3.514	1.84E-06
CCL2	2.23	2.04E-06
ERG	5.112	2.09E-06
CXCL3	3.109	3.39E-06
CXCL2	3.057	3.39E-06
Ccl2	2.497	4.70E-06
WNT3A	2.287	4.90E-06
PLG	4.065	5.59E-06
TET2	2.828	6.97E-06
KLF4	3.272	9.16E-06
IL27	4.527	1.10E-05
CEBPD	2.434	1.21E-05
MIF	4.191	1.32E-05
TLR7	5.145	1.35E-05
SMAD1	2.3	4.74E-05
GLI1	2.211	1.02E-04
FOXA3	−2.093	1.06E-04

*The z-score indicates the predicted activation state of a transcription factor or key regulator: z ≤ −2 indicates relevant inhibition (shown in green), z ≥ 2 indicates relevant activation (shown in red). The p-value indicates significant enrichment of the genes downstream of a regulator (p < 0.001 was considered statistically significant)*.

### Overlap in inflammatory processes between human and experimental NASH

**Figure 4:**
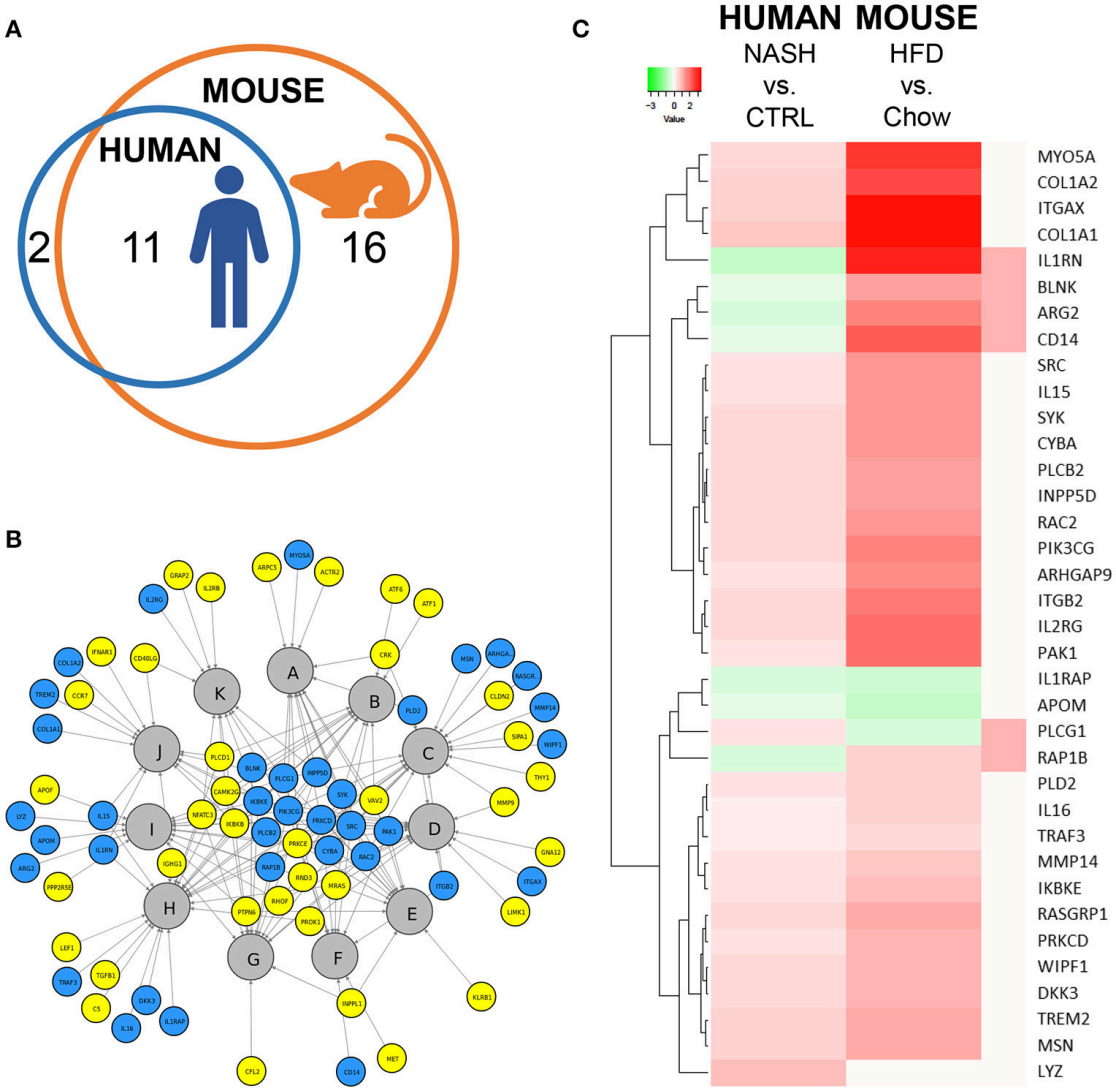
Representation of human key molecular pathways and associated genes in HFD-fed Ldlr^−/−^.Leiden mice. **(A)** Venn diagram to visualize the overlap in canonical pathways between human NASH biopsies (red circle) and Ldlr^−/−^.Leiden mice (blue circle). **(B)** Network visualization of overlapping canonical pathways (gray nodes, A: Fcγ Receptor-mediated Phagocytosis in Macrophages and Monocytes, B: PI3K Signaling in B Lymphocytes, C: Leukocyte Extravasation Signaling, D: IL-8 Signaling, E: Natural Killer Cell Signaling, F: Macropinocytosis Signaling, G: B Cell Receptor Signaling, H: Role of Macrophages Fibroblasts and Endothelial Cells in Rheumatoid Arthritis, I: Production of Nitric Oxide and Reactive Oxygen Species in Macrophages; J: Dendritic Cell Maturation; K: iCOS-iCOSL Signaling in T Helper Cells) and the associated significantly expressed genes in human NASH (blue and yellow nodes). The blue nodes represent genes that were also regulated in the Ldlr^−/−^.Leiden mouse, the yellow nodes represent genes that were not significantly regulated in the Ldlr^−/−^.Leiden mouse. **(C)** Heatmap visualization of genes underlying the common pathways that are regulated in both human NASH biopsies and HFD-fed Ldlr^−/−^.Leiden mice relative to their respective controls. Red color indicates upregulated genes and green color indicates downregulated genes. The rightmost column shows the genes that do not share their direction of regulation between human and mouse in red.

Next, we investigated in more detail whether these NASH-associated molecular inflammatory processes are similar in human and murine NASH, and to what extent the individual genes are similarly regulated. To evaluate the overlap of canonical inflammatory pathways between human and murine NASH, pathways were integrated using an overlay Venn-diagram. Thirteen inflammatory pathways were significantly regulated in human NASH and 27 inflammatory pathways were significantly regulated in murine NASH. Interestingly, a large part-−11 out of 13 pathways—of the human inflammatory processes were also regulated in HFD-fed Ldlr^−/−^.Leiden mice (Figure 4A), including several macrophage-related pathways such as “Production of Nitric Oxide and Reactive Oxygen Species in Macrophages,” “Macropinocytosis Signaling,” and “Fcγ Receptor-mediated Phagocytosis in Macrophages and Monocytes” but also pathways related to other inflammatory cell types, such as “Dendritic Cell Maturation,” “B Cell Receptor Signaling,” and “Natural Killer Cell Signaling” (Table 3). Next, we investigated the differentially expressed genes underlying the shared pathways in human NASH and identified which of these were also regulated in the HFD-fed Ldlr^−/−^.Leiden mouse (Figure 4B). We then evaluated the conformity in direction of these genes that were differentially regulated in both human and murine NASH (i.e., upregulation or downregulation relative to control) (Figure 4C) and found that 83% of the shared genes (in either human or murine NASH) changed in the same direction in human and mouse NASH.

**Table 3:** Overlap analysis of significantly regulated pathways in human and murine NASH.

		**Human NASH vs. CTRL**	**Ldlr^−/−^.Leiden mouse HFD vs. chow**
**No**.	**Canonical Pathway**	**−log(*****p*****-value)**	**−log(*****p*****-value)**
**PATHWAYS REGULATED BOTH IN HUMAN NASH AND MURINE NASH**			
1	Fcγ Receptor-mediated phagocytosis in macrophages and monocytes	5.44	12.5
2	PI3K signaling in B lymphocytes	3.77	7.2
3	Leukocyte extravasation signaling	3.56	6.29
4	IL-8 signaling	3.05	9.48
5	Natural killer cell signaling	2.95	5.82
6	Macropinocytosis signaling	2.87	4.78
7	B Cell receptor signaling	2.54	4.89
8	Role of macrophages, fibroblasts, and endothelial cells in rheumatoid arthritis	2.53	5.74
9	Production of nitric oxide and reactive oxygen species in macrophages	2.36	7.98
10	Dendritic cell maturation	2.05	6.5
11	iCOS-iCOSL signaling in T helper cells	2.04	5.35
**PATHWAYS REGULATED ONLY IN MURINE NASH**			
1	CD28 signaling in T helper cells	1.82	5.51
2	Crosstalk between dendritic cells and natural killer cells	1.61	4.05
3	T cell receptor signaling	1.53	3.02
4	Acute phase response signaling	1.43	6.84
5	PKCθ signaling in T lymphocytes	1.43	4.58
6	IL-10 signaling	1.28	4.56
7	IL-12 signaling and production in macrophages	1.18	4.02
8	LPS/IL-1 mediated inhibition of RXR function	0.981	7.85
9	Altered T cell and B cell signaling in rheumatoid arthritis	0.833	5.41
10	Th1 and Th2 activation pathway	0.505	4.19
11	NF-κB signaling	0.375	5.21
12	Th2 pathway	0.26	3.11
13	T helper cell differentiation	0.254	3.57
14	Toll-like receptor signaling	0.247	6.11
15	Inflammasome pathway	0.242	3.42
16	Th1 Pathway	N/A	3.98
**PATHWAYS REGULATED ONLY IN HUMAN NASH**			
1	Chemokine signaling	2.16	2.44
2	LPS-stimulated MAPK signaling	2.16	2.99

*Significance of enrichment for canonical pathways is indicated by –log(p-value)*.

**Figure 5:**
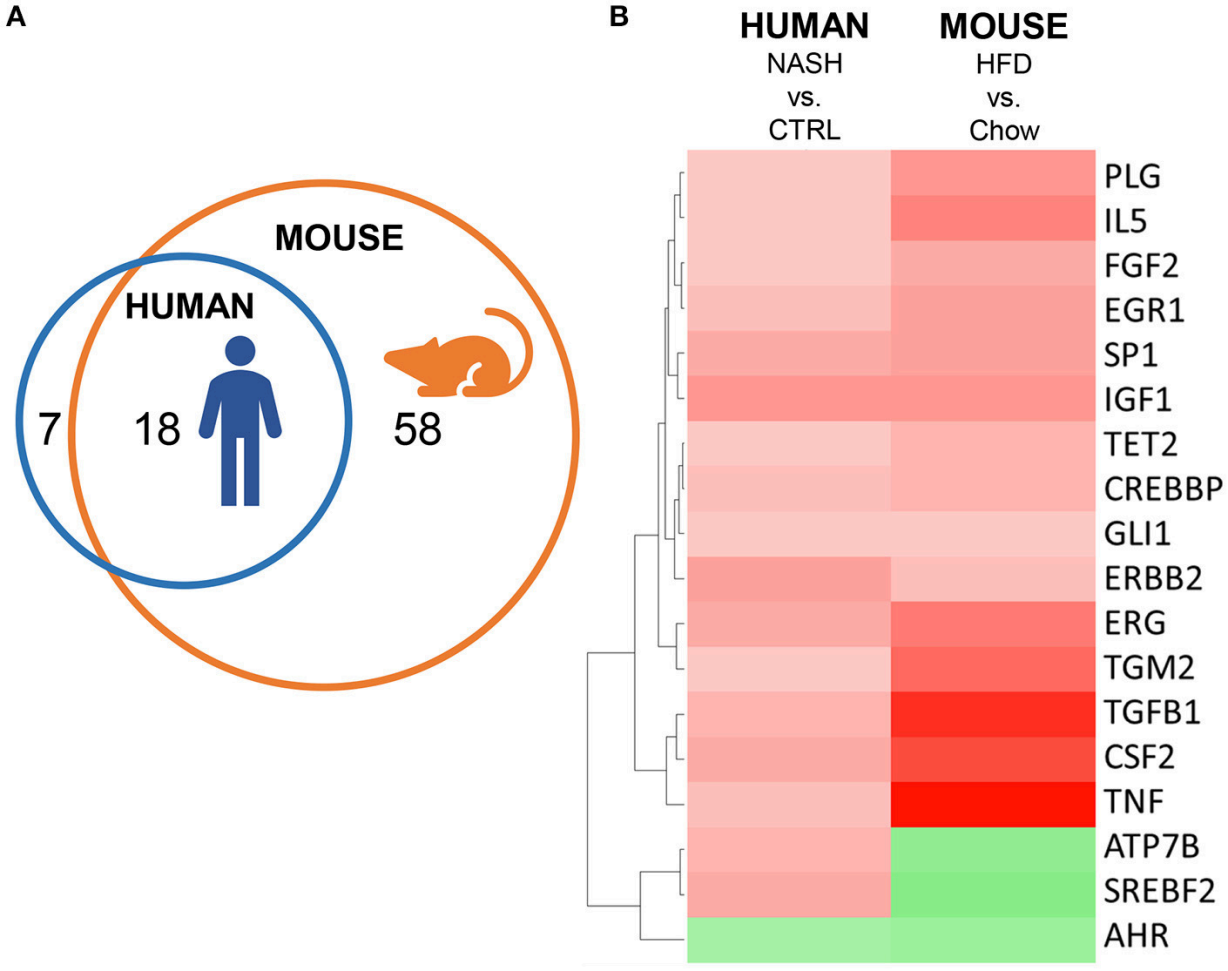
Representation of human inflammation-related upstream regulators in HFD-fed Ldlr^−/−^.Leiden mice. **(A)** Venn diagram to visualize the overlap in upstream regulators between human NASH biopsies (red circle) and Ldlr^−/−^.Leiden mice (blue circle). **(B)** Heatmap visualization of direction of regulation (Z-score). Red color indicates activated upstream regulator and green color indicates inhibited upstream regulator.

Finally, we investigated the overlap of upstream regulators between human and mouse NASH using an overlay Venn-diagram (Figure 5A). A total of 25 inflammation-related upstream regulators was significantly regulated in human NASH, and 76 inflammation-related upstream regulators were significantly regulated in HFD-fed mice. Of the 25 inflammation-related factors that were regulated in human NASH, 18 were also affected in HFD-fed Ldlr^−/−^.Leiden mice, including the pathways downstream of the cytokines TNF, TGFB1 and CSF2 which were activated in both human and murine NASH. Analysis of the direction of regulation, i.e., predicted activation or predicted inhibition of the upstream regulator, showed that the majority of the overlapping upstream regulators was regulated in the same direction in Ldlr^−/−^.Leiden mice as in NASH patients (Figure 5B) supporting the translational value of the experimental model conditions.

## Discussion

This study defines the major molecular inflammatory pathways and key regulators in human NASH using gene profiling data from liver biopsies, and provides evidence that the major molecular responses in humans can be replicated experimentally in a diet-inducible NASH model, the HFD-fed Ldlr^−/−^.Leiden mouse.

Studies on human gene expression in NASH are scarce, typically include only a small number of patients, and datasets are not always made publicly available (Moylan et al., 2014; Arendt et al., 2015; Teufel et al., 2016; Lefebvre et al., 2017). Typically, the patients used therein are drawn from a patient population with known heterogeneity (Machado and Diehl, 2016; Younossi et al., 2016) for instance due to variations in disease etiology, ethnicity, gender, lifestyle and dietary habits. Herein we used an open access gene profiling dataset (Ahrens et al., 2013; Teufel et al., 2016) and, despite these limitations, such as a limited number of samples, were able to identify a number of significantly regulated inflammatory pathways and upstream regulators, indicating that these may be key inflammatory processes that are common in many NASH patients. Many of the identified pathways describe processes to do with inflammatory cells, such as “Leukocyte Extravasation Signaling” and “Chemokine Signaling” which are required for the infiltration of immune cells into the liver, one of the diagnostic criteria for NASH (Kleiner et al., 2005). Several were related to the activity of macrophages (e.g., “Production of Nitric Oxide and Reactive Oxygen Species in Macrophages,” “Macropinocytosis Signaling,” and “Fcγ Receptor-mediated Phagocytosis in Macrophages and Monocytes”), a cell type that is believed to play an important role in the inflammatory response in NASH (Itoh et al., 2013; Alisi et al., 2017) and is thus considered a promising target for treatment (Tacke, 2017). Immune cell infiltration in general, and macrophage responses in specific, can be the result of various pro-inflammatory stimuli (for instance cholesterol or gut-derived LPS; Heymann and Tacke, 2016), and may therefore represent a downstream phenomenon in NASH that can be the result of various disease-inducing pathways, providing a potential explanation for its involvement across the heterogenous patient population.

To provide meaningful information on disease development, disease mechanisms, or effects of new therapeutic interventions, it is critical that a pre-clinical model not only reflects histopathological features of human disease, but also recapitulates human (patho)physiological processes on the molecular level. We found that the vast majority of inflammatory pathways and upstream regulators that were significantly modulated in human NASH were also significantly modulated in the Ldlr^−/−^.Leiden mouse (11 out of 13 pathways and 18 out of 25 upstream regulators), indicating that this model recapitulates the key inflammatory processes of human NASH.

Inbred mouse strains such as the Ldlr^−/−^.Leiden mouse are genetically homogenous and allow study of NASH development under standardized experimental conditions as reported (Morrison et al., 2016; Schoemaker et al., 2017). Furthermore, mice liver samples are collected at a relatively uniform stage of disease development depending on the time of high-caloric diet feeding (Liang et al., 2014; Arnoldussen et al., 2017), while human samples show more varying histopathology within a disease stage. In reflection of this high degree of homogeneity, substantially more genes were differentially expressed relative to healthy control in the Ldlr^−/−^.Leiden mice than in the human samples, and we also found a larger number of pathways and upstream regulators significantly modulated in mice (with a higher level of significance). For instance, we observed regulation of several T-cell related pathways in HFD-fed Ldlr^−/−^.Leiden mice that were not significantly regulated in the human NASH samples (e.g., “Th1 and Th2 Activation Pathway,” “T Helper Cell Differentiation,” “T Cell Receptor Signaling”). Although much remains unknown about the role of T cells in the development and progression of NASH, alterations in this immune cell population have been reported for NASH patients (Inzaugarat et al., 2011), specifically in more advanced disease stages (i.e., fibrosis score >F2; Gadd et al., 2014). The lack of regulation of T cell-related pathways observed in the current study may be reflective of the relatively low presence of fibrosis in this patient cohort (mostly F1). In general, pathways that were found to be significantly enriched in mice but not in patients may constitute pathways that are more variably enriched in human disease (e.g., in a subset of NASH patients but not in others—resulting in an average expression level that does not pass the threshold for enrichment). Conversely, there were 2 pathways that were regulated in human NASH but not in the Ldlr^−/−^.Leiden mouse; “LPS-stimulated MAPK Signaling” and “Chemokine Signaling.” In both pathways it is specifically the MAPK expression which is distinctive in human compared to mouse liver tissue. Since mice are housed under pathogen-free conditions (SPF) it is amenable that activation of MAPK by LPS is more likely to be observed in the human situation.

Besides phenotypically readily apparent subtypes of NASH patients, such as lean vs. obese patients (Kumar and Mohan, 2017) or diabetic vs. non-diabetic patients (Puchakayala et al., 2015), recent efforts on subtyping NAFLD/NASH patients on the basis of their serum metabolome (Alonso et al., 2017; Iruarrizaga-Lejarreta et al., 2017) have revealed that patients may also be classified on the basis of their molecular disease patterns and have provided insight into molecular pathways that may be impaired in some but not other patients (i.e., synthesis of S-adenosylmethionine). A similar analysis in the Ldlr^−/−^.Leiden mouse revealed that on the metabolome level this model reflects a substantial proportion of NAFLD/NASH patients (Morrison et al., 2017) but the underlying disease mechanisms remain unclear. Further exploration on the gene expression level may shine light on different disease etiologies and the molecular pathways involved in various disease-inducing mechanisms, as well as their divergent representation in the NASH patient population. However, such studies require large cross-sectional gene expression datasets of well-characterized and uniformly graded biopsies of different stages that allow classification of patients into molecular subtypes.

Given this large diversity of disease-inducing mechanisms in human NASH, it is unlikely that one single preclinical model for NASH will reflect the entire spectrum of underlying molecular responses observed in patients. However, individual models may recapitulate specific aspects of the spectrum of molecular responses seen in humans and can be of value to study that particular mechanism (Morrison et al., 2015; Iruarrizaga-Lejarreta et al., 2017; Zimmer et al., 2017). At the same time, one must always be aware of the limitations of the model employed. The Ldlr^−/−^.Leiden model for instance, while mimicking many aspects of human NASH (e.g., pathophysiology, histology, underlying molecular processes; Liang et al., 2014; Morrison et al., 2016; Mulder et al., 2016; van Koppen et al., 2018), is unsuitable to study interventions that require a functioning LDL receptor (Zimmer et al., 2017). Although many different (diet-inducible) experimental models of NASH have been reported (reviewed elsewhere Takahashi et al., 2012), the vast majority of these models has only been characterized on the pathophysiological and histological level and thus their value in the study of human disease processes remains unclear and requires further investigation.

Altogether, this study defines, to our knowledge for the first time, the key molecular inflammatory responses in biopsies of NASH patients and demonstrates that these are reflected in HFD-fed Ldlr^−/−^.Leiden mice. Comparative gene profiling approaches may help to better estimate the translational value of preclinical models for the NASH population in general, or specific subgroups of NASH patients in particular. Models that are validated on the molecular level against human disease pathways and key regulators of inflammation, constitute important tools to evaluate new therapeutics that target these pathways.

## Author contributions

MM, RK, AvK, RH, and LV: Study concept and design; MM, and LV: Writing the manuscript; MM, and LV: Analysis and interpretation of data; MM, RK, AvK, RH, and LV: Critical revision of manuscript.

### Conflict of interest statement

The authors declare that the research was conducted in the absence of any commercial or financial relationships that could be construed as a potential conflict of interest.
